# Stay close, but not too close: aerial image analysis reveals patterns of social distancing in seal colonies

**DOI:** 10.1098/rsos.230269

**Published:** 2023-08-09

**Authors:** J. P. A. Hoekendijk, A. Grundlehner, S. Brasseur, B. Kellenberger, D. Tuia, G. Aarts

**Affiliations:** ^1^ NIOZ Royal Netherlands Institute for Sea Research, 1790AB Den Burg, The Netherlands; ^2^ Wageningen University and Research, 6708PB Wageningen, The Netherlands; ^3^ Wageningen Marine Research, Wageningen University and Research, 1781AG Den Helder, The Netherlands; ^4^ Department of Ecology and Evolutionary Biology, Yale University, New Haven, CT, USA; ^5^ Ecole Polytechnique Fédérale de Lausanne (EPFL), 1950 Sion, Switzerland

**Keywords:** nearest neighbour distance, density, remote sensing, *Phoca vitulina*, *Halichoerus grypus*, herding behaviour

## Abstract

Many species aggregate in dense colonies. Species-specific spatial patterns provide clues about how colonies are shaped by various (a)biotic factors, including predation, temperature regulation or disease transmission. Using aerial imagery, we examined these patterns in colonies on land of two sympatric seal species: the harbour seal and grey seal. Results show that the density of grey seals on land is twice as high as that of harbour seals. Furthermore, the nearest neighbour distance (NND) of harbour seals (median = 1.06 m) is significantly larger than that of grey seals (median = 0.53 m). Avoidance at small distances (i.e. social distancing) was supported by spatial simulation: when the observed seal locations were shuffled slightly, the frequency of the smallest NNDs (0–25 cm) increased, while the most frequently observed NNDs decreased. As harbour seals are more prone to infectious diseases, we hypothesize that the larger NNDs might be a behavioural response to reduce pathogen transmission. The approach presented here can potentially be used as a practical tool to differentiate between harbour and grey seals in remote sensing applications, particularly in low to medium resolution imagery (e.g. satellite imagery), where morphological characteristics alone are insufficient to differentiate between species.

## 1. Introduction

Colony formation is an ecological trait that occurs in many animal taxa. The process of colony formation is driven by various biotic and abiotic costs and benefits [1]. Potential benefits include protection from predators (i.e. predator swamping), thermoregulation, mating success, increased foraging efficiency and information transfer [2,3]. Benefits associated with colony forming are case-specific and by no means universal among different species [4,5]. Colony formation also has costs. Two of these—namely increased competition for resources and an increased risk of pathogen transmission—are considered inevitable [4–6]. The interplay between these various costs and benefits influences the size of colonies (i.e. the tendency to stay with many conspecifics, but not too many), and may lead to distinct fine-scale spatial patterns (i.e. ‘stay close, but not too close’). These patterns are a potentially valuable tool for remote sensing applications: the distinct spatial distribution patterns may be used to identify species, which opens new possibilities to use lower resolution imagery (e.g. satellite imagery with 31 cm per pixel resolution) that is otherwise insufficient to differentiate species based on morphological characteristics of single individuals.

**Figure 1.**
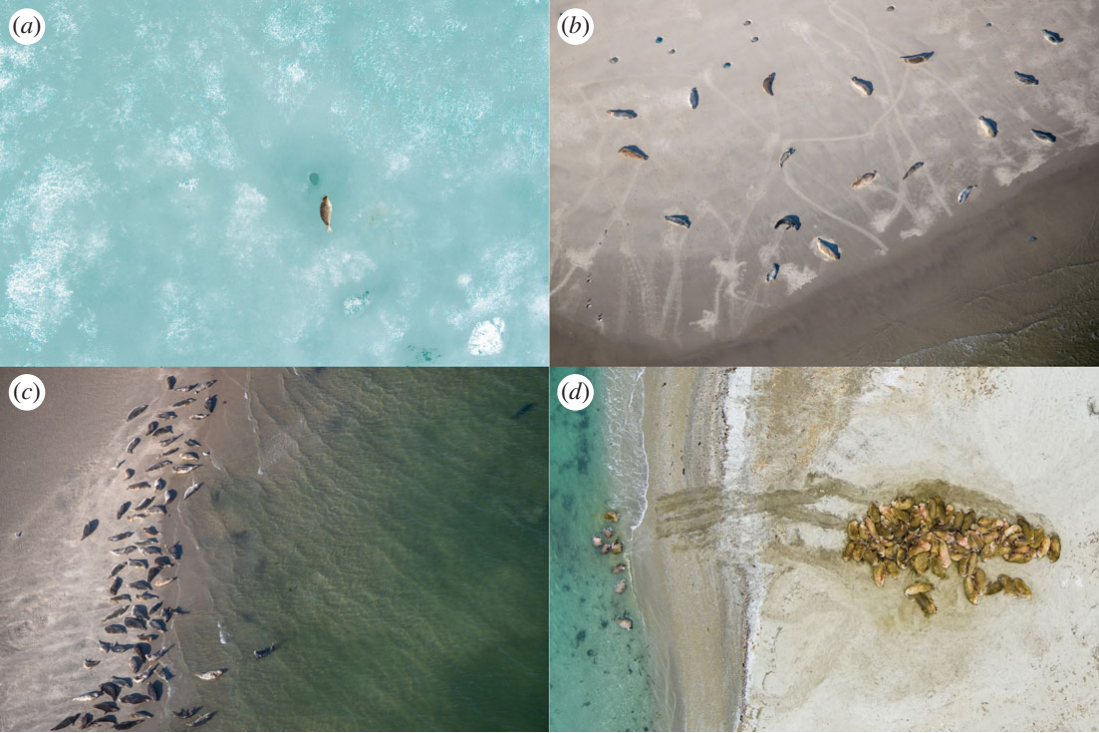
Fine-scale haul-out patterns of pinnipeds. Haul-out patterns of pinnipeds show high variation. Some species haul out solitarily, such as ringed seals (*a*), while others—such as harbour seals (*b*) and grey seals (*c*)—haul out in colonies while preserving some distance from conspecifics. Finally, some species—such as walruses (*d*)—may cluster together without any distance between individuals. Photos (*a*) and (*d*) by Eelke Folmer (Aeria).

Pinnipeds (i.e. seals, sea lions, fur seals and walruses) occupy the boundary between the marine and terrestrial realm. They forage in a marine environment, but depend on land or ice to rest, moult and pup [7]. Many pinniped species tend to cluster together when hauling out on land, regularly forming large aggregations. Previous research has shown that these colonies can increase pup survival in southern sea lions *Otaria byronia* [8], while for elephant seals *Mirounga leonina*, pup mortality increases with higher densities in colonies [9]. For harbour seals *Phoca vitulina*, alertness increases with group size [10,11], which suggests that scanning for approaching danger could be another important benefit and driver for colony forming in pinnipeds. On the other hand, a commonly observed cost that limits pinniped group size and density is competition for haul-out space, which might result in agonistic behaviour [12–18]. This competition for space is potentially fiercer on land than on ice, as the sea-ice is generally more widely available than suitable haul-out sites on land. The various costs and benefits of colony forming have resulted in a wide range of—potentially species-specific—fine-scale haul-out patterns (figure 1).

In the southern North Sea and Dutch Wadden Sea, grey seals *Halichoerus grypus* and harbour seals *Phoca vitulina* are considered sympatric species (i.e. having an overlapping habitat and distribution) [19–21]. Both species haul out on intertidal flats, sand banks and beaches [22]. Grey seals generally haul out on the highest sandbanks, which are less exposed to tidal and weather conditions, while harbour seals most often use sandbanks that are only available during low tide. Especially during the pupping season, grey seals avoid tidal haul-out sites, as their pups need to remain on land for several weeks to moult and shed their birth coat (i.e. the lanugo) before going to sea [23,24]. On the contrary, harbour seal pups moult their lanugo *in utero* and can swim within hours after birth, which allows them to utilize lower sandbanks and intertidal flats even during the breeding season. Consequently, harbour seals have more suitable haul-out sites available during the pupping season than grey seals. The preference of grey seals for higher grounds seems general and is also observed outside the breeding season, most notably during the moult when they aggregate in groups reaching over a thousand individuals. Grey seals tend to undertake longer foraging trips and have longer resting times compared with harbour seals [22], which might explain their preference for higher haul-out sites safe from tidal conditions. Despite these differences, there is occasional overlap, where grey and harbour seals are observed mixed together on a haul-out site.

The species-specific differences in haul-out behaviour most likely play an important role in explaining their population dynamics. While both species have historically been hunted extensively in the Wadden Sea, the breeding system of the grey seal may render this species more vulnerable. For grey seals, this led to their extinction in this area in the Middle Ages [23]. Protective measures and legislation in the UK in the early twentieth century allowed neighbouring grey seal populations to recover and subsequently recolonize the Wadden Sea in the 1980s. Fuelled by this immigration [24], the grey seal population in the Wadden Sea has grown to over 9000 individuals (counted during moult). Harbour seals on the other hand, are more mobile (even with pups) and difficult to approach and are more likely to escape into the water when facing threats. Compared with grey seals, they were therefore less vulnerable to historic hunting, which is reflected in an abundance estimate of 40 000 individuals in the Wadden Sea in 1900 [25,26], despite centuries of hunting [27]. However, due to a more extensive use of firearms and industrial pollution, the harbour seal population decreased dramatically to around 4500 individuals in 1960 [28]. After that, recovery was limited due to pollution [29] and two outbreaks of the phocine distemper virus (PDV) in 1988 and 2002. During both outbreaks, the population was reduced to approximately 50% [30–32]. Despite these massive reductions in the recent past, the harbour seal is currently the most abundant seal species in the Wadden Sea.

The influence of (a)biotic factors—such as pathogen transmission, availability of preferred haul-out sites and requirements related to phenology or social cohesion—may result in species-specific fine-scale haul-out patterns within grey and harbour seal colonies. To examine the fine-scale spatial haul-out patterns of grey and harbour seals, we analyse measurements of densities and spatial distances between individual seals at various haul-out sites in The Netherlands, using high-resolution aerial imagery. We then show that the observed densities and distances are species-specific and differ significantly between the two species, with harbour seals keeping larger distances from conspecifics than grey seals. By shuffling the observed distributions through spatial simulations, we then show that both species avoid getting too close to conspecifics and that distribution of inter-individual distances vary greatly between the species. This finding has implications to understand pinnipeds behaviour, but also could be used as a proxy for large-scale species identification. Indeed, when detecting, counting and analysing sympatric behaviour of seals in lower resolution (satellite) imagery, one could use the inter-individual distances to characterize the species of the group. This could become a valuable tool to aid in species identification based on satellite images of inaccessible regions, such as the Arctic.

## 2. Methods

### 2.1. Data collection on fine-scale distribution

The Dutch government has commissioned the collection of aerial images as part of the national inspection of land-use change (i.e. land registry ‘Kadaster’), but some of these images also contain seal haul-out sites. Surveys were conducted once per year, during February–June, between 2016 and 2019. The images were georeferenced (projection: Amersfoort Rijksdriehoek; EPSG:28992) and have a resolution of either 7.5 cm (2019) or 10 cm (2016–2019). The distribution of individual grey (*N* = ∼80) and harbour seals (*N* = ∼250) tracked with GPS loggers was used to determine the exact location of seal colonies on land (i.e. haul-out sites). Aerial images that overlapped with these tracked animals were selected for visual inspection in QGIS (v. 3.10) and the colonies were categorized as grey seal (i), harbour seal (ii) or mixed (iii) colonies. All images that contained seals were then selected for analysis and each individual seal was manually labelled, by drawing a polygon following the outline of each seal, using the Picterra software suite (www.picterra.ch). The annotations were then exported as georeferenced spatial polygon shapefiles. Mixed colonies were excluded from further analysis.

### 2.2. Nearest neighbour distance and density estimation

To determine inter-animal distances and examine fine-scale spatial patterns, the polygons (each one corresponding to an individual seal) were analysed in the statistical software R (v. 1.4.1106) [33] (for the complete R-code, see electronic supplementary material, S4). For each photographed haul-out site a distance matrix was created using the *gDistance* function from the *rgeos* R-package [34], which contained the distances (in metres) between the edges of all polygons within the haul-out site. Assuming that the spatial position of every individual seal represents an independent decision, the smallest distance for each polygon—representing the nearest neighbour distance (NND)—was extracted from the distance matrix. The mean and median NND were calculated for both species. Since the mean NND is highly influenced by outliers, we tested if there was a significant difference in the median between the NNDs of grey seals and harbour seals by fitting a 0.5 quantile regression model (package *quantreg*, function *rq*, [35,36]) to the data, where ‘species’ was included as factor variable.

**Figure 2.**
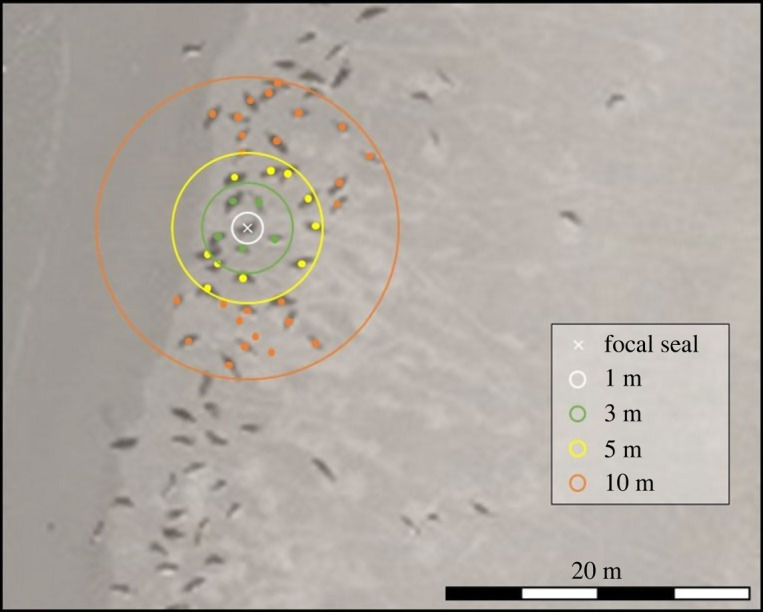
Visualization of the density analysis. For a focal seal (white cross) all neighbouring seals were counted within a 1 (white), 3 (green), 5 (yellow) and 10 m (orange) radius. This was repeated for every seal.

Additionally, as a measure for density, we plotted circles with increasing radii (1, 3, 5 and 10 m) around the centre point of a focal seal, and counted how many neighbouring seal centre points were present within each circle (figure 2). This was repeated for every seal. To test if there were significant differences between the densities of grey seals and harbour seals, a generalized linear model (GLM) was fitted to these count data, assuming a negative binomial error distribution to allow for possible over- or under-dispersion (package *MASS*, function *glm.nb*, [37]), and including ‘species’ as factor variable. This analysis was repeated for all radii (1, 3, 5 and 10 m) separately.

### 2.3. Social distancing analysis

Hauled-out seals group together, but within these aggregations, seals may choose to maintain a small distance between individuals. To examine this social distancing, we simulated spatial arrangements of the colony via spatial perturbation of the seals positions: the annotated seals (i.e. the polygons) were semi-randomly moved to a new location within the colony while maintaining the heterogeneous spatial density on the haul-out site, and the resulting simulated NNDs were compared with the original NNDs. To do this, the spatial distribution of seals on each individual haul-out site was defined by estimating a spatial kernel density (package *spatstat*, function *densityfun*, [38]) based on the original observed distribution of all polygons. The bandwidth used for the kernel density was the average cross-validated bandwidth determined per haul-out site (package *spatstat*, function *bw.diggle*, [38]). For each polygon, a new location was sampled based on the kernel around the centre of each polygon. The orientation of the seal was preserved. This way, the observed densities and colony forming behaviour of the seals was mimicked and the sampling space was limited to represent the original space in which the seals were distributed. The shuffled polygons often overlapped (35% for grey seals, 14% for harbour seals). As it is uncommon for either seal species to lay on top of each other, overlapping polygons were rotated (1-degree increments). If the overlap was not resolved after rotation, the polygon was moved slightly in a random direction with 10 cm increments until the overlap was resolved. Additionally, two alternative approaches for dealing with overlapping polygons were also analysed and can be found in electronic supplementary material, S1.

We investigated whether seals keep a (small) distance from one another (i.e. social distancing), by comparing the NNDs of the non-shuffled polygons (i.e. the original observations) with the shuffled NNDs. This was tested for both species separately, by comparing the proportion of the polygons lying within 25 cm of each other in the observed and shuffled dataset. This threshold distance of 25 cm was chosen to prevent any bias introduced by potential imprecise annotation caused by the image resolution (7.5 or 10 cm per pixel). The statistical testing was done by fitting a GLM with binomial error distribution to the data (with the number of NNDs < 25 cm and >25 cm as ‘successes’ and ‘failures’, respectively) and observed/shuffled as factor variable.

## 3. Results

As the national land registry focused on human terrestrial use, therefore neglecting tidal sites, relatively few seal haul-out sites were recorded in Dutch waters. After visual inspection of the aerial images that overlapped with the GPS tracking data, a total of 11 haul-out sites were found (electronic supplementary material, S3). Based on the GPS tracking data, two of these sites were identified as grey seal colonies and six as harbour seal colonies. Three sites containing mixed groups of harbour and grey seals were excluded from further analysis, to allow for a comparison between species. The images of the grey seal sites were collected in March, during the moult. Most of the harbour seal images were collected in February–May, during the feeding season. However, one harbour seal haul-out site was photographed in June, during the pupping season of harbour seals, and multiple mother/pup pairs were visible. As mother/pup pairs tend to stay close together, and would bias the nearest neighbour distance results, this site was excluded from the analysis. A detailed comparison of this site with non-pupping harbour seal haul-out sites can be found in electronic supplementary material, S2.

**Figure 3.**
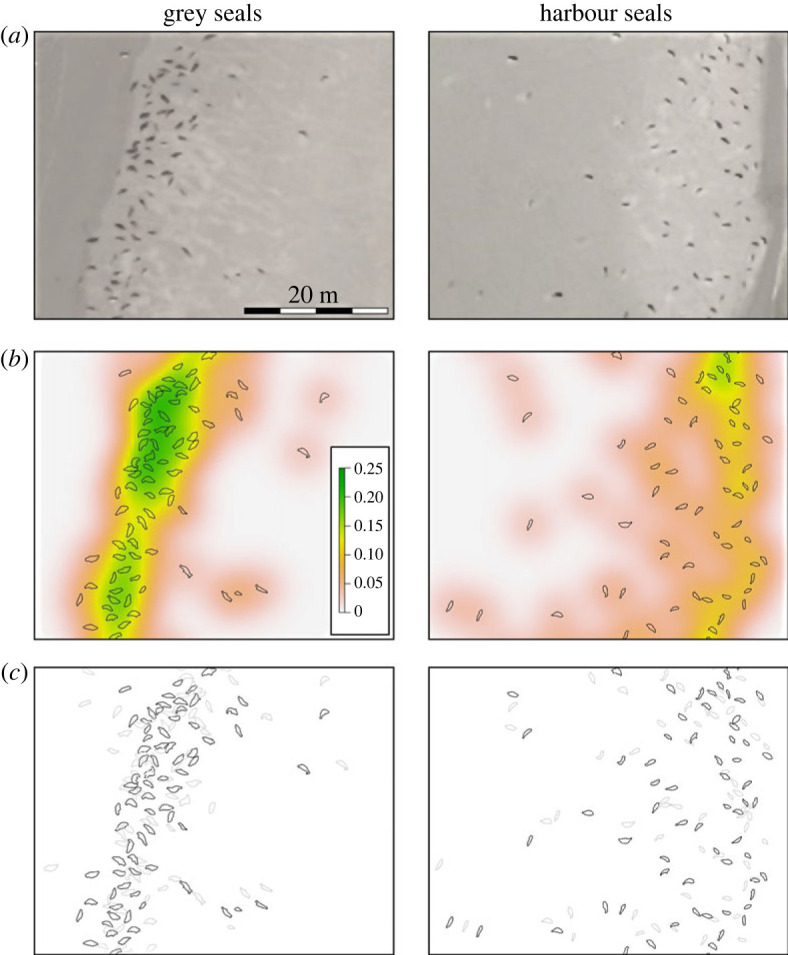
Density kernel and randomization. (*a*) Example of original aerial image of grey seals (left) and harbour seals (right). (*b*) Visualization of density kernels, with annotated seals, of the same region as (*a*). (*c*) Original distribution (black) and shuffled distribution (light grey), of the same region as (*a*) and (*b*).

In the selected images, a total of 1574 harbour seals (February–May) and 3299 grey seals (March) were found and annotated. The mean NND for harbour seals was 1.62 m, and 1.15 m for grey seals. The median NND for harbour seals (1.06 m) was twice as large as that of grey seals (0.53 m). This difference was significant (table 1). In line with Graves *et al*. [39] we also calculated 25% quantiles, which were 0.32 and 0.49 m for grey and harbour seals, respectively. These quartiles also significantly differed between species (*t*-value 8.32539, *p* < 0.001). The patterns observed for grey and harbour seals are consistent across the different haul-out sites (figure 3). For all harbour seal haul-out sites, the median and interquartile range are larger than those of the grey seal sites.

**Table 1.** Summary statistics (*t*-test median based on quantile regression) comparing the NND of grey and harbour seals.

species	*N*	sites	mean (m)	median (m)	*t*-test median
grey seal	3299	2	1.15	0.53	*t* = 18.3
harbour seal	1574	5	1.62	1.06	*p*-value < 0.001

The number of neighbouring polygons present within different radii (1, 3, 5 and 10 m) around a focal polygon differed significantly between grey and harbour seals for all radii, with grey seals having roughly two times higher density than harbour seals (table 2).

**Table 2.** Haul-out densities of grey seals (*Hg*) and harbour seals (*Pv*), for different radii around a focal polygon. The results of the GLsuM for all radii are provided, as well as the mean and median number of individuals and the 95% confidence interval (CI) for both species.

radius (m)	mean		95% CI		median		GLM *(Hg* versus *Pv)*
	*Hg*	*Pv*	*Hg*	*Pv*	*Hg*	*Pv*	
1	1.86	0.63	1.81–1.91	0.59–0.68	2	0	*z*-value = −29.86
							*p*-value < 0.0001
3	7.23	3.30	7.07–7.38	3.15–3.46	7	3	*z*-value = −34.06
							*p*-value < 0.0001
5	14.01	6.97	13.73–14.29	6.68–7.27	15	5	*z*-value = −32.97
							*p*-value < 0.0001
10	32.34	18.34	31.74–32.95	17.62–19.10	33	15	*z*-value = −28.48
							*p*-value < 0.0001

**Figure 4.**
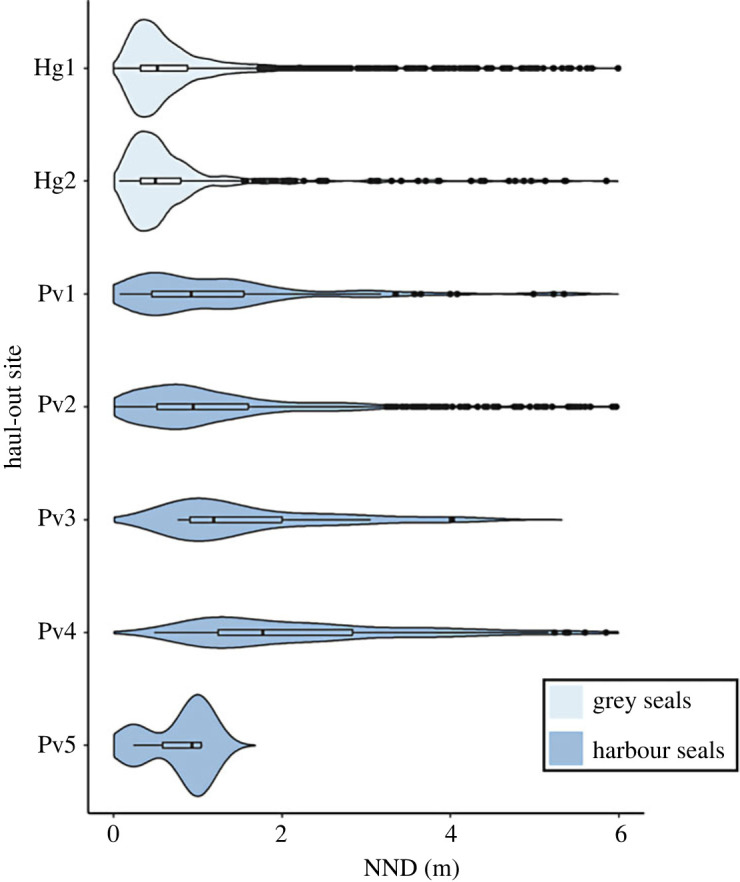
Distribution NNDs per site. Violin plots of NNDs for each grey seal (light blue) and harbour seal (dark blue) haul-out site.

**Figure 5.**
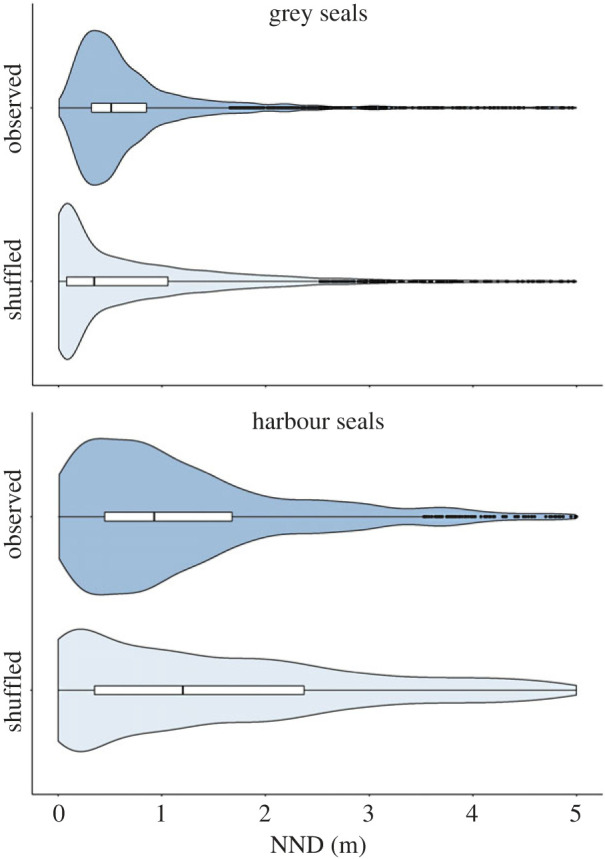
Distribution NNDs. Violin plots of NNDs for both the observed (dark blue) and the shuffled distribution (light blue), for grey seals (top) and harbour seals (bottom).

After the random displacements of polygons (see Methods section; figure 4), we found that for both grey and harbour seals the shuffled distribution of NNDs comprised a higher frequency of both smaller and larger NNDs, while fewer NNDs at intermediate distances were observed with respect to the NNDs of the original observations (figure 5).

**Figure 6.**
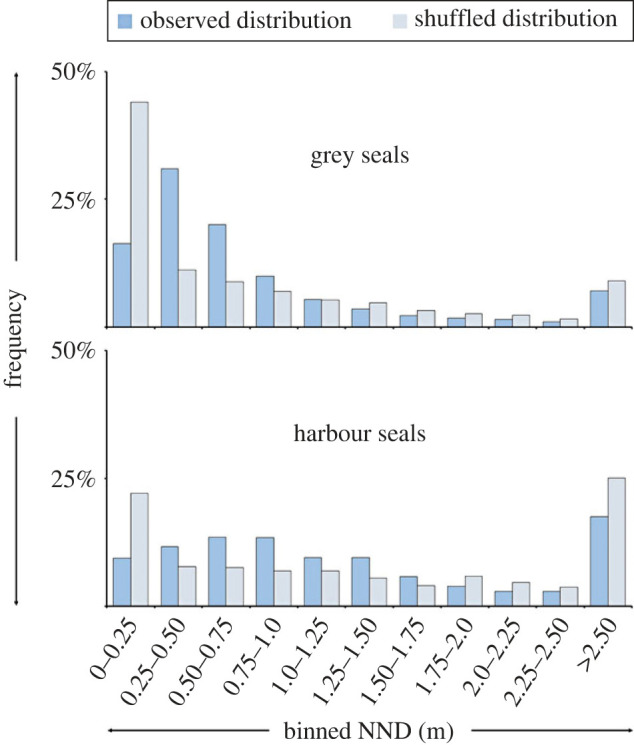
Distribution of observed and shuffled NNDs. Distribution of the NNDs in 0.25 m bins, for grey seals (top) and harbour seals (bottom). In the shuffled distribution (light blue), the smallest NNDs are more common than in the observed distributions (dark blue).

Compared with the observed distribution, the occurrences of the smallest NNDs (0–25 cm) increased for both species after the shuffling. For grey seals, 16% (537 out of 3299) of the observed seals are within 25 cm of their nearest neighbour, while this proportion increases to 44% after shuffling (1453 out of the 3299). This difference is statistically significant (GLM, *z*-value = 23.8, *p*-value < 0.001). For harbour seals, only 9% (148 out of 1574) observed individuals are within 25 cm of their neighbour, while after shuffling this is increased to 22% (348 out of the 1574), which is also statistically significant different (GLM, *z*-value = 9.5, *p*-value < 0.001) (figure 6). This indicates that both seal species avoid the immediate proximity (less than 25 cm) of their neighbour.

## 4. Discussion

Pinniped behaviour is strongly governed by their phenology and shows great seasonal variation. During approximately nine months of the year, seals spend most of their time at sea, while regularly coming back to shore to rest. However, during breeding and moulting they spend more time on land and seal numbers at haul-out sites are generally higher. Furthermore, seasonal variation in behaviour within colonies has also been recorded: during the breeding season of harbour seals for instance, the level of alertness is lower [10,40,41]. This seasonal variation can potentially also affect the fine-scale distribution (and the NNDs reported in this study). However, the aerial images available for this study did not allow to examine all periods (i.e. feeding, breeding and moult) for both seal species. The only images available for grey seals were collected in March during the moult, while for harbour seals, images from both the feeding season (February–May) and pupping season (June) were available. This allowed for the comparison between the two species in spring. Additionally, we provide an example of seasonal differences in haul-out patterns for harbour seals in and outside the breeding season (electronic supplementary material, S2). Here we found that pupping harbour seals display both significantly lower densities and median NNDs than non-pupping harbour seals. This can be explained by mother/pup pairs keeping more distance from conspecifics during nursing, while the pup stays in very close proximity to its mother. Additional survey data is required to further study seasonal variations and interspecific variation between colonies.

The manual processing of the aerial imagery as presented in our study is labour-intensive and time consuming. However, thanks to the recent rapid developments in the field of computer vision, it could be possible to utilize automated detection algorithms to reduce the time required to label individual animals in newly collected imagery (e.g. [42,43]). The images and annotations collected in our study can be used to train such an algorithm for hauled-out grey and harbour seals in the Wadden Sea.

Like most other pinnipeds, harbour and grey seals haul out in groups. In our study, harbour seals keep more distance—i.e. display greater NNDs—from conspecifics than grey seals do. Furthermore, harbour seals occur in significantly lower densities at haul-out sites compared with grey seals, despite being considerably smaller than (male) grey seals and therefore requiring less space. Both grey and harbour seal avoid close (less than 25 cm) contact.

No other studies on harbour seal NNDs were found. Studies on fine-scale haul-out patterns for grey seals are scarce and limited to the breeding season, whereas our grey seal images were collected during the moult. During the breeding season, grey seals form harems. Female grey seals have been estimated to haul out within 8 m of another female [44], which is similar to an estimated NND of 5–10 m between multiple grey seal mother–pup pairs [7]. Both studies examined grey seals hauled out on ice, where suitable habitat is generally less scarce than on land. On land, an NND of 6.07 m was reported for breeding females [45]. The lower NNDs reported in our study could potentially be explained by the lack of aggression among males and among nursing females guarding their pups, a behaviour which is commonly observed during the breeding season [46].

**Figure 7.**
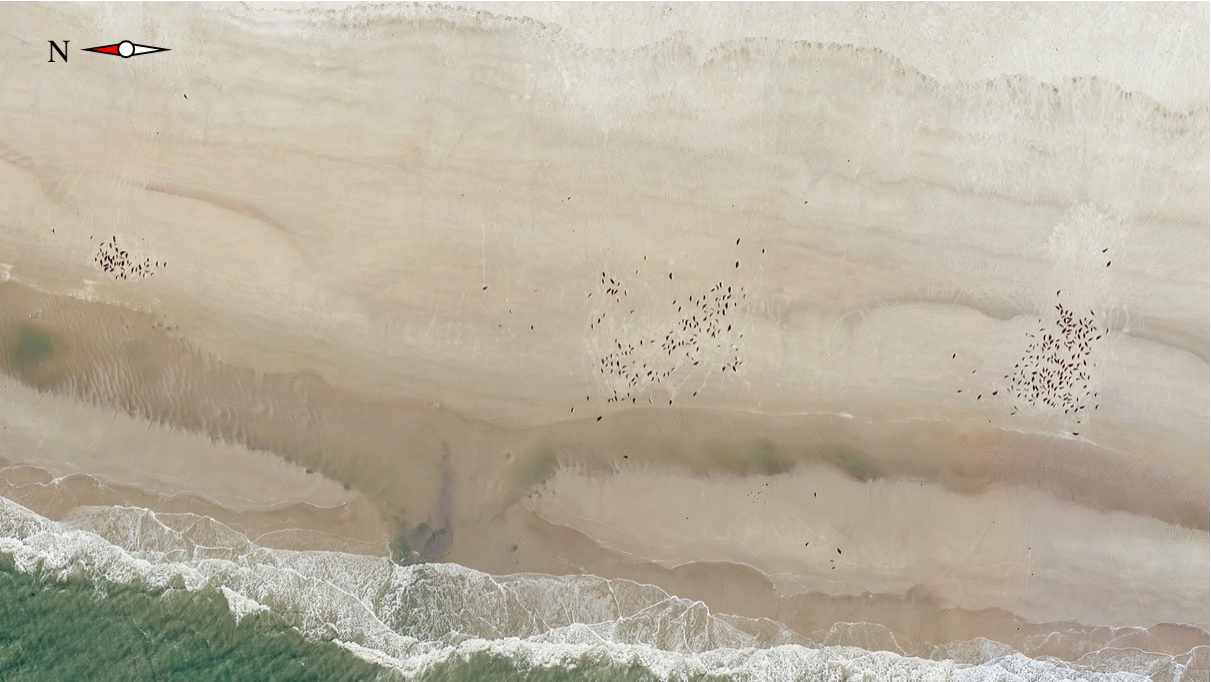
Hauled-out grey seals. Aerial image of hauled-out grey seals. Even when suitable haul-out space is not limiting, grey seals often tend to cluster together in our study.

The underlying mechanisms driving the observed differences in the fine-scale haul-out patterns of grey and harbour seals remain unclear. Although still speculative at this point, the two universal costs of colony formation—increased competition for resources and an increased risk of pathogen transmission—provide potential insights. Due to the preference of grey seals in the Wadden Sea area for relatively higher haul-out sites [23,24], suitable haul-out space for grey seals is more limited than for harbour seals. Consequently, competition for space is higher, which could potentially explain the smaller NNDs and higher haul-out densities of grey seal. However, on one of the two grey seal haul-out sites in this study, haul-out space does not seem to be a limiting factor, yet grey seals often tend to cluster in tight groups (figure 7). Both grey seal haul-out sites are relatively high and also available during high tide, allowing seals to move up during incoming tide.

With a larger group size, the prevalence of pathogens (including parasites) increases [47–49]. When facing emerging pathogens, both affected individuals and healthy individuals can mitigate infection risks by altering their behaviour and reducing their level of interaction (i.e. their sociality) as a precautionary measure [48,50]. Therefore, pathogen occurrence not only limits group size, but also the level of sociality within a group [51]. The effectiveness of this response was also illustrated in humans during the outbreak of the SARS-CoV-2 virus, as many countries implemented social distancing measures, which significantly reduced the transmission rate [50,52]. Because seals haul out in close proximity of many other individuals and have high contact rates, they are particularly vulnerable to infectious diseases [53]. The two PDV outbreaks in 1988 and 2002 serve as an example of this: harbour seal populations were reduced by up to 50%, whereas grey seals remained relatively unharmed by the same virus. It is possible that the observed differences in fine-scale haul-out patterns between the two species reflect an evolutionary response to pathogen occurrence. Interestingly, this behaviour—where individuals alter their level of sociality in response to an emerging pathogen (i.e. on an ecological timescale)—has been observed in other social animals [50], such as mule deer *Odocoileus hemionus hemionus* [54], wild house mice *Mus musculus domesticus* [55] and social insects [56]. For Caribbean spiny lobsters, it has been shown that attraction to conspecifics has decreased in a region with a higher pathogen occurrence [48,57], which is suggested to be an evolutionary response. Although our findings confirm the existence of social distancing in harbour and grey seals, it cannot be concluded whether or not it is an evolutionary response to limit pathogen transmission, because no data was available from before or during these outbreaks.

The observed fine-scale haul-out patterns of grey and harbour seals are species-specific, which is particularly interesting for remote sensing applications. In The Netherlands, grey and harbour seals are sympatric, young and subadult grey seals are of similar size as adult harbour seals, and both species haul out throughout the year. Consequently, it is challenging to differentiate the two species in remote sensing imagery with an insufficient resolution to identify the species based on morphological characteristics. Whereas the resolution of imagery used in our study is 7.5 and 10 cm per pixel, the resolution of the highest resolution of commercially available satellite imagery is currently 31 cm per pixel. Although this allows for the detection of individual seals (e.g. [58]), it is impossible to differentiate between harbour and grey seals based on morphological characteristics alone. For this type of low-resolution imagery, additional variables such as seal phenology (e.g. seasonality) and habitat characteristics (e.g. height of haul-out sites) can aid in the identification of grey and harbour seal colonies to the species level. Our findings suggest that spatial patterns within a colony could provide another tool to differentiate between these species. This approach has potential for the characterization of colonies in remote and inaccessible regions such as the polar regions, where satellite images are routinely available.

## Data Availability

The data used in this study are open-source and publicly available. Code and data associated with this study can be obtained at https://dataportal.nioz.nl/doi/10.25850/nioz/7b.b.3d. The data are provided in electronic supplementary material [59].
